# Regulation of human glioma cell apoptosis and invasion by miR-152-3p through targeting DNMT1 and regulating NF2: MiR-152-3p regulate glioma cell apoptosis and invasion

**DOI:** 10.1186/s13046-017-0567-4

**Published:** 2017-08-01

**Authors:** Jin Sun, Xinhua Tian, Junqing Zhang, Yanlin Huang, Xiaoning Lin, Luyue Chen, Shizhong Zhang

**Affiliations:** 1 Department of Neurosurgery, Zhujiang Hospital Southern Medical University, National Key Clinical Specialty, Engineering Technology Research Center of Education Ministry of China, Guangdong Provincial Key Laboratory on Brain Function Repair and Regeneration, Guangzhou, Guangdong 510282 China; 2 Department of Neurosurgery, Zhongshan Hospital, Xiamen University, Xiamen, Fujian 361004 China

**Keywords:** Glioblastomas, NF2, DNMT1, miR-152-3p, Demethylation

## Abstract

**Background:**

MiRNAs are involved in aberrant DNA methylation through regulation of DNA methyltransferases (DNMTs) in the pathogenesis and progression of glioblastomas (GBM). MiR-152-3p was down-expressed in human malignancies, and served as a tumor suppressor. Neurofibromatosis type 2 (NF2) was significantly decreased in GBM tissues with a high level of methylation. However, the link between miR-152-3p, DNMT1 and methylation of NF2 in GBM is not clearly established. This study was conducted to detect the mechanism between miR-152-3p, DNMT1 and NF2 in GBM.

**Methods:**

The levels of DNMT1 and NF2 expression were studied by qRT-PCR, Western blot, immunofluorescence, and immumohistochemical staining. Methylation in the promoter region of NF2 was detected by methylation-specific PCR and bisulfate genomic sequencing PCR. Cell proliferation was examined by Cell-Counting Kit-8 and 5-ethynyl-2′-deoxyuridine assay, and cell invasion was evaluated by transwell assay. Flow cytomery and Hoechst staining were used to analyze cell apoptosis. A dual luciferase system was used to confirm the relationship between miR-152-3p and DNMT1.

**Results:**

Methylation of NF2 and DNMT1 was markedly increased, and miR-152-3p was downregulated in GBM tissues and glioma cells. Both knockdown of DNMT1 and overexpression miR-152-3p showed that demethylation activated the expression of NF2. Furthermore, miR-152-3p directly targeted DNMT1. Both miR-152-3p overexpression and DNMT1 knockdown significantly induced cell apoptosis and inhibited invasive activity. This was also observed after NF2 overexpression.

**Conclusions:**

These results indicated that miR-152-3p can inhibit glioma cell proliferation and invasion activities by decreasing DNMT1. The restoration of miR-152-3p may have therapeutic application in the treatment of GBM.

**Electronic supplementary material:**

The online version of this article (doi:10.1186/s13046-017-0567-4) contains supplementary material, which is available to authorized users.

## Background

Glioblastoma, also known as glioblastoma multiforme (GBM), is the most common and aggressive type of brain tumour in adults and accounts for about 69% of all gliomas [1, 2]. GBM is characterized by extensive infiltration throughout the brain parenchyma, robust angiogenesis and necrogenesis, intense resistance to apoptosis, and genomic instability [3]. The current standard conventional treatment for GBM includes neurosurgery, radiotherapy and chemotherapy [4]. However, these aggressive treatments are not effective in controlling the disease, and the prognosis remains dismal, with a median survival of 12–18 months [5]. Therefore, a better understanding of the molecular mechanism of invasiveness and proliferation of GBM cells is important in the development of a more effective therapeutic target in GBM.

The neurofibromatosis type 2 (NF2) gene is a tumour suppressor gene, and its inactivation is important in the development of NF2-associated tumours. The product of the *NF2* gene is termed neurofibromin 2 (NF2) protein, also known as merlin or schwannomin. The protein functions as a linker between transmembrane proteins and the actin cytoskeleton and regulates cytoskeleton remodelling [6]. There is growing evidence that demonstrates its critical role in governing cell survival, motility and invasiveness. Recent work on the tumour suppressor function of NF2 has shown that it is dramatically downregulated in malignant gliomas, resulting in enhanced proliferation of glioma cells, and that it plays a pivotal role in tumorigenesis [6–8].

Many factors are involved in the regulation of NF2 activity, such as gene mutation, expression and phosphorylation [9]. It is well recognized that the silencing of tumor suppressors and oncogenes can result from DNA methylation. Disruption of methylation has been observed in GBM. DNA methylation at the 5-position of cytosine is initiated and maintained by catalysis of DNA methyltransferases (DNMTs) [10]. Aberrant DNMT expression has been shown to facilitate tumorigenesis and development [11]. Such epigenetic changes are potentially reversible and therefore are considered promising targets for anti-cancer treatments. Indeed, DNA-demethylating drugs have been approved by the Food and Drug Administration (FDA) as a treatment for myelodysplastic syndromes and myelogenous leukemia [6, 7]. DNMT1 is a major enzyme responsible for DNA methylation and heavily contributes to the methylation of NF2 in benign meningioma cells and leptomeningeal cells [12]. However, the link between the methylation of NF2 and DNMT1 in GBM is not well understood.

MicroRNAs (miRNAs) are endogenously expressed, short noncoding RNAs of 20–23 nucleotides that target messenger RNA (mRNA). They increase the molecular heterogeneity of GBM and function as micro-modulators in the migration and invasion of GBM cells. Therefore, the potential role of miRNAs in the treatment of GBM has become widely recognized. Growing evidence indicates that miRNAs are involved in aberrant DNA methylation through regulation of DNMTs, contributing to tumorigenesis and tumor development. MicroRNA-29 (miR-29) reverts aberrant methylation by targeting DNMT3a and DNMT 3b [13]. MiR-185 is involved in ovarian cancer through targeting DNMT1 [14]. However, the correlation between miRNAs and DNMT1 in GBM, and their role in the development of GBM is largely unknown.

In this study, we investigated the role of miRNAs in the regulation of DNMT1 and NF2 expression, and in the resultant invasiveness of GBM cells. This is the first study to observe that miR-152-3p negatively regulates the expression of DNMT1 and invasiveness of GBM cells. Additionally, the miRNA was found to be involved in the expression of NF2 via methylation. Conclusively, our results suggest a new molecular mechanism underlying regulation of the development of GBM by NF2.

## Methods

### Tissue samples from GBM patients

GBM tissues and adjacent tissues were collected from patients who underwent curative resection at the Xiamen University Affiliated Zhongshan Hospital, Fujian, China. Fresh samples were stored at −80 °C in a freezer. The study was approved by the Ethical Committee of Xiamen University Affiliated Zhongshan Hospital, and consent was obtained from all participating patients.

### Cell culture and transfection

The human normal glial cell line, HEB, and the human glioma cell lines U251, U87, T98-G and A172 were obtained from the American Type Culture Collection. The cells are routinely cultured in Dulbecco’s modified Eagle media (Hyclone) with 10% FBS (GIBCO, Life Technologies, Helgerman Court, MD, USA), 100 units/ml penicillin and 100 mg/ml streptomycin in a humidified atmosphere at 5% CO_2_ and 37 °C.For transfection, U251 cells were seeded at 5 × 10^5^ cells/well in 6-well plates. The cells were transfected with the DNMT1 siRNA (5′ UGUUAAGCUGUCUCUUUCCAAGGAAAGAGACAGCU UAACAGA 3′) or negative control (NC) siRNA with Lipofectamine 2000 (Invitrogen) based on the manufacturer’s instruction. Overexpression of miR-152-3p and NF2 were achieved by transfecting the cells with miR-152-3p mimics (miR-152-3p) and NF2-overexpressing vector obtained from Genepharma (Shanghai, China) and Vipotion (Guangzhou, China), respectively. The siRNAs, miR-152-3p and their associated control were obtained from GenePharma, Shanghai, China. About 48 h after transfection, the cells were collected for apoptosis determination and expression analysis by qRT-PCR, Western bot and methylation-specific PCE.

### Quantitative real-time PCR (qRT-PCR)

The expression of NF2, DNMT1 mRNA and miR-152-3p was determined with real-time quantification PCR. Total RNA was extracted from U251 cells or tissue samples using the TRIzol reagent (Takara) according to the manufacturer’s protocol. Total RNA (1 μg) was reverse transcribed with a Bestar™qPCR RT kit (Applied Biosystems) in a 20 μl PCR reaction. The levels of the mRNAs and miRNA were quantified by real-time PCR (Stratagene Mx3000P) using DBI Bestar® SybrGreen qPCR master Mix. To determine the levels of NF2 and DNMT1 mRNA, RT-PCR was performed with the following primers: NF2, forward: CCCCCAACTCCCCTTTCC ,reverse: AGCCCTTTAGCCCCCCTG and DNMT1, forward: GACCCACGAAAGCCACC, reverse: CACCTCACAGACGCCACA. The β-actin gene was used as an internal control. To quantify the level of primary miRNA, qRT-PCR was performed using primary miR-152-3p-specific primers (forward: ACACTCCAGCTGGGTCAGTGCATGACAG, reverse: CTCAACTGGTGTCGTGGAGTCGGCAATTCAGTTGAGCCAAGTT). The miRNA expression was normalized to the small nuclear RNA U6. All quantitative qRT-PCRs were performed in triplicate using the 2-ΔΔCt method.

### Methylation specific PCR

Methylation Specific PCR (MSP) was performed with 4 μl/40 ng bisulfite-modified DNA and 25 μl PCR mixture with 1.8× PCR buffer, 5 mM MgCl2, 100 pmoldeoxynucleotide triphosphates, primers (100 pmol per reaction) and one unit of Taq Platinum (Invitrogen). PCR amplification was conducted at 95 °C for 3 min, followed by 4 cycles (94 °C for 1 min, 60 °C for 30 s, and 72 °C for 45 s), and then followed by 28 amplification cycles (94 °C for 1 min, 56 °C for 1 min, and 72 °C for 45 s). Finally, the elongation was conducted at 72 °C for 4 min. For parallel quality control, a plasmid containing methylated NF2 sequence and water without DNA template were used as positive and negative control, respectively. The sequences of PCR primers specific for NF2 are given in Table 1. Finally, MSP products were analyzed using 2% agarose gel electrophoresis with ethidium bromide.

**Table 1:** Primers for Methylation Specific PCR of NF2

Primers	Sequence(5′- 3′)	Product Length(bp)
NF2-M F	TGCGTTGAAATTTAATAATTTTACG	303
NF2-M R	CCAAACTAAAATACAATAACGCGAT	
NF2-UF	TGTGTTGAAATTTAATAATTTTATGA	303
NF2-UR	CCAAACTAAAATACAATAACACAAT	

### Bisulfite Sanger sequencing

A total of 500 ng of genomic DNA extracted from GBM and malignant glioma cell lines was bisulfite converted using a MethylCode™ Bisulfite Conversion Kit (Applied Biosystems, USA). The *NF2* promoter was amplified by PCR with *Taq* DNA Polymerase (Invitrogen, USA). The primer sequence was designed using Methyl Primer Express™ Software v1.0 (Applied Biosystems, USA). Sanger sequencing was performed on the PCR products. Five single molecules were sequenced for each sample.

### Western blot assay

After washing with PBS, the cells were lysed and centrifuged at 14000 g for 10 min at 4 °C. The protein concentrations were determined using a BCA protein assay kit. Equal amounts of whole-cell lysates were separated on 10% SDS-PAGE, then transferred to PVDF membranes (Millipore, Danvers, MA, USA). After blocking with 5% skimmed milk in TBST at room temperature (RT) for 2 h, the membranes were probed with primary antibodies (anti-NF2 [ab88957] and DNMT1[ab19905], 1:1500 dilution; anti-GAPDH, 1:2000 dilution; Abcam) overnight at 4 °C, and then incubated with the appropriate secondary antibodies. Finally, the probed proteins were visualized using enhanced chemiluminescence solution.

### Annexin V assay

The apoptosis of U251 cells was quantified using a FITC-labeled AnnexinV/propidium iodide (PI) Apoptosis Detection kit (Beyotime, Beijing, China) according to the instructions. Flow cytometric analysis was performed immediately after staining using a flow cytometer (Beckman, USA). Cells in early stage apoptosis werepositive for Annexin V, whereas cells in late stage apoptosis were positive for both AnnexinV and PI positive.

### EDU assay and Hoechst staining

Cell proliferation was detected using a 5-ethynyl-2′-deoxyuridine assay kit (Ribobio, Guangzhou, China; EdU), according to the manufacturer’s instructions. Briefly, U251 cells transfected with pcDNA-NF2, DNMT1 siRNA, miR-152-3p, or their respective controls were exposed to 50 μM EdU for 2 h. After collection, the cells were fixed in 4% formaldehyde for about 20 min and then permeabilized with 0.5% Triton X-100 for about 10 min at RT. After washing with PBS, the cells were treated with 200 μL Apollo ® reaction cocktail for 10 min and then permeabilized with 0.5% TritonX-100. In addition to detection with flow cytometry, the cells suspended in PBS were stained with 100 μL Hoechst 33,342 (5 μg/mL) and visualized with a fluorescent microscope (Olympus, Japan).

### Luciferase reporter assay

The luciferase reporter assay was performed with the Dual-Luciferase® Reporter (DLR™) Assay System (Promega) according to the manufacturer’s instructions. In brief, U251 cells were plated at a density of 2 × 10^4^ cells per well in 24-well plates for 24 h. After incubation, 4 μg of plasmids were cotransfected with pMIR-DNMT1–3′-UTR-wt/mut and 2 ng of pRL-TK using Lipofectamine 2000 (Invitrogen). The cells were collected after about 48 h in culture for luciferase assays using the Luciferase Reporter Assay system (GloMax) according to the manufacturer’s protocol. The assay was performed in triplicate.

### Cell-Counting Kit-8 proliferation assay

U251 cells, pretreated with pcDNA-NF2, DNMT1 siRNA or the miR-152-3p, were seeded at 2000 cells/well in 96-well plates for 24 h. Cell viability was analyzed using a Cell-Counting Kit-8 (CCK-8) proliferation assay kit following the manufacturer’s instruction. Absorption intensity was measured at 450 nm using a microplate spectrophotometer (TECAN, Australia).

### Immunohistochemistry

Specimens were provided by the Xiamen University Affiliated Zhongshan Hospital, Fujian, China. The study was approved by the Ethical Committee of Xiamen University Affiliated Zhongshan Hospital, and consent was obtained from all participating patients. Surgically resected specimens were fixed in formalin and embedded in paraffin. Expression of NF2 and DNMT1 were evaluated using immunohistochemistry (IHC). After the tissue sections (4 μm thickness) were deparaffinized and rehydrated, microwave antigen retrieval was conducted in citrate buffer. After cooling, endogenous peroxidase was produced with 3% hydrogen peroxidase for 5 min at RT. Then the slides were blocked with 5% goat serum for 30 min at RT and incubated overnight at 4 °C with primary antibodies against 2 μg/ml NF2 or 10 μg/ml DNMT1 (Abcam). After being washed in PBS with 0.05% Tween 20, the sections were incubated with secondary antibody for 30 min at RT. After subsequent washes with PBS, DAB was applied for visualization of the indicated proteins.

### Immunofluorescence staining

The treated cells were rinsed with PBS and then fixed with 4% paraformaldehyde in PBS for 1 h. After re-rinsingwith PBS, the cells were permeabilized with 0.1% Triton X-100 for 2 min on ice, and washed with PBS twice. Immunofluoresence analysis was conducted using specific primary antibodies against rat DNMT1 (Abcam, Cambridge, MA, USA) and against NF2 (Abcam, Cambridge, MA, USA). The cells were subsequently incubated with DAPI detecting liquid for 10s at RT followed by microscopic observation, and results were recorded.

### Determination of the invasiveness of GBM cells

The invasiveness of U251 cells was assessed using Matrigel transwells (BD Biocoat Matrigel Invasion Chamber). U251 cells, pretreated with pcDNA-NF2, DNMT1-siRNA or the miR-152-3p, were seeded at 5× 10^5^ cells/well in 6-well plates for 12 h. Subsequently, the cells were cultured with serum free medium for 12 h and then seeded at 2 × 10^4^ cells in 100 μl serum-free media per transwell coated with matrigel. In the lower chamber, 700 μl complete medium with 10% FBS was added. After 48 h, the cells were fixed with 4% paraformaldehyde and stained with crystal violet. Invasiveness was quantified by counting the number of invaded cells from six fields.

### Statistical analysis

Data were presented as mean ± SD. Statistical differences were analyzed by two-sided Student’s t-test. *P* values less than 0.05 were considered significant.

## Results

### NF2 and miR-152-3p were down-regulated, DNMT1 was upregulated in GBM cell lines and tumour tissues

**Fig. 1:**
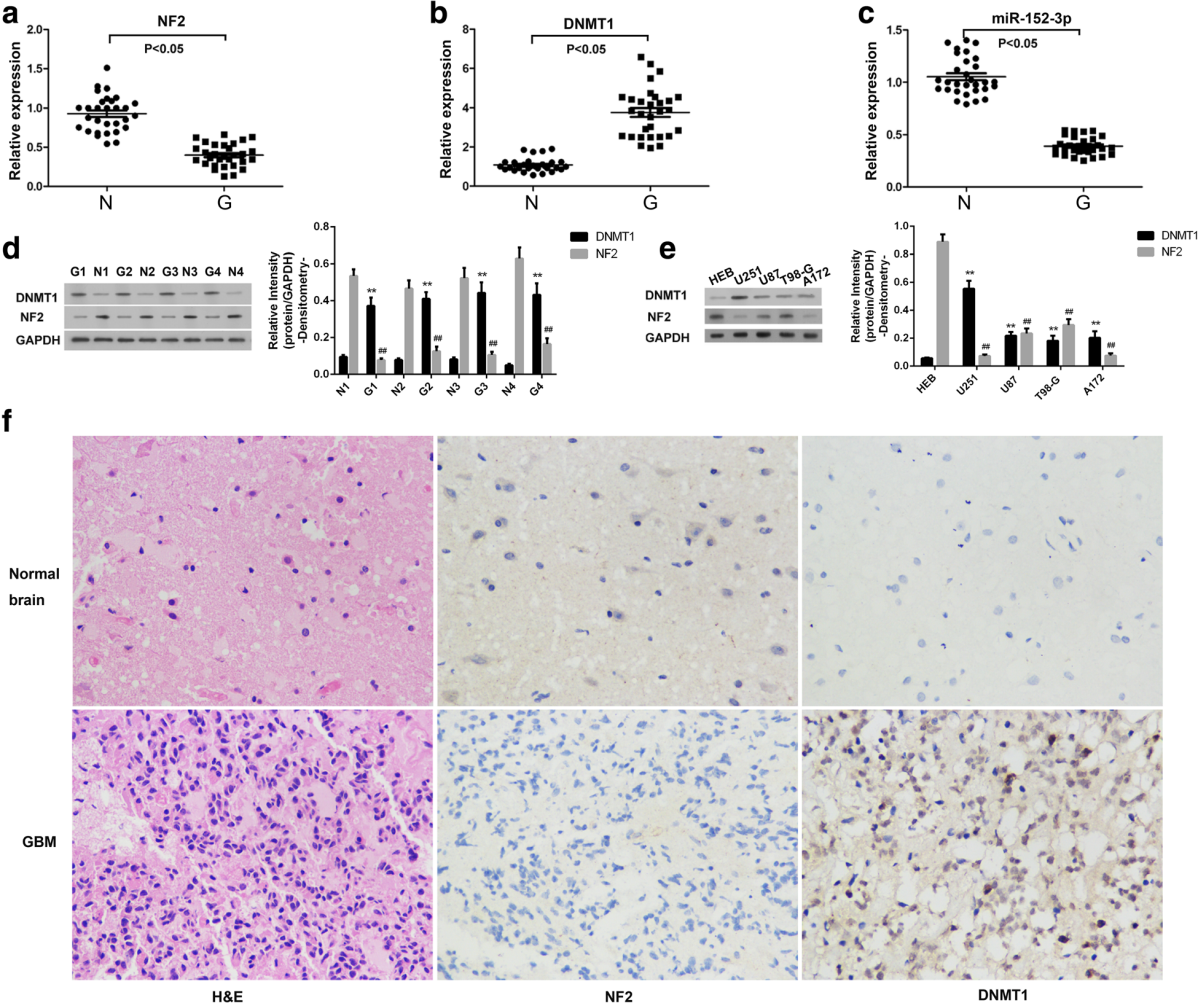
The expression of NF2, DNMT1, and miR-152-3p in GBM and malignant glioma cell lines. Normal tissue and GBM were collected to determine mRNA expression of NF2 (**a**), DNMT1 (**b**) and miR-152-3p (**c**), as well as protein expression of NF2 and DNMT1 (**d**). N, normal brain; G, GBM. Protein expression of NF2 and DNMT1 in HEB, U251, U87, TG-G and A172 cells was evaluated (**e**). Typical histologic sections of normal tissue and GBM, as well as immunostaining for NF2 and DNMT1 in normal brain and GBM are shown (**f**). Magnitude 400×. **, *P* < 0.01 indicated the NF2 expression compared with the expression of control groups. ##, *P* < 0.01 indicated the DNMT1 expression compared with the expression of control groups

NF2 is widely recognized as an important contributor to GBM. Thus, we investigated its levels of mRNA and protein expression. As shown in Fig. 1a, NF2 mRNA in GBM was significantly decreased compared with normal brain. As DNMT1 plays an important role in the regulation of the expression of various proteins, its expression was evaluated to determine whether it was involved in the downregulation of NF2. As shown in Fig. 1b, we observed that DNMT1 mRNA was significantly increased in GBM compared with matched normal brain control tissue. Because miRNAs are widely accepted as a promising therapy for many diseases, especially tumours, miR-152-3p was investigated by RT-PCR in this study. It was noted that miR-152-3p was significantly decreased in GBM (Fig. 1c). Protein expression levels of NF2 and DNMT1 were determined by Western blot. As shown in Fig. 1d, protein expression of NF2 in GBM was significantly decreased and that of DNMT1 was significantly increased compared with control tissues. These observations were confirmed by IHC detection (Fig. 1f). HEB cells are human normal glial cell line, whereas U251, U87, T98-G and A172 cells are human glioma cells. In these cell lines, we observed that DNMT1 and NF2 were significantly increased and decreased, respectively, in glioma cells compared with HEB cells (Fig. 1e). These data indicated that NF2 might contribute to the development of GBM and that the process might be mediated by DNMT1 and miR-152-3p.

### Hypermethylation of NF2

**Fig. 2:**
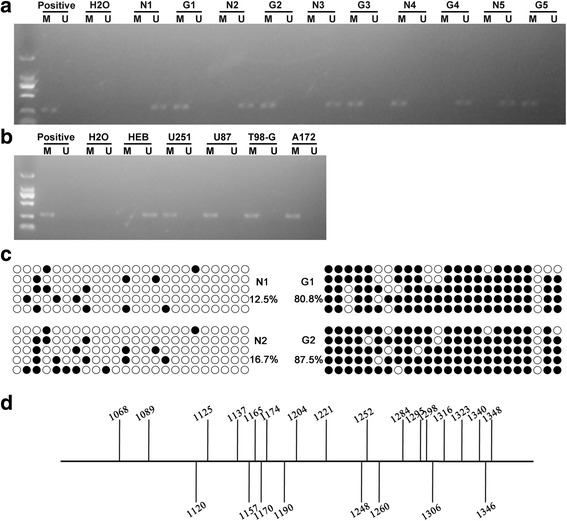
Hypermethylation of NF2 in GBM and malignant glioma cell lines. The methylation status of NF2 was detected in normal brain and in GBM (*n* = 5). N, normal brain; G, GBM (**a**). The methylation status of NF2 was detected in HEB, U251, U87, T98-G and A172 cell lines (**b**). Increased *NF2* promoter methylation in GBM brain samples compared with normal brain samples was detected by bisulfite Sanger sequencing (**c**); the exact methylation sites are indicated (**d**)

Given that DNMT1 methylation is critical in DNMT1 regulation of protein expression, the methylation status of NF2 in GBM was assayed in 12 GBM samples versus normal tissue control samples. As shown in Fig. 2a, hypermethylation of NF2 was observed in 10 (83.3%) of 12 GBM. Unexpectedly, this was also observed in 2 (16.7%) of 12 normal tissues (data not shown). These results indicated that the incidence of NF2 hypermethylation in GBM was markedly higher than in normal tissues. Based on the high correlations observed between NF2 hypermethylation and GBM, the methylation status of NF2 was potentially involved in GBM. Subsequently, HEB, U251, U87, T98-G and A172 cells were used to determine the methylation status of NF2, to investigate of the correlation between NF2 methylation and GBM. As shown in Fig. 2b, hypermethylation of NF2 was noted in U251, U87, T98-G and A172 cells, but not in HEB cells. In order to further explore whether the methylation sites are located on CpG site sites, which are believed to be associated with decreased gene expression, bisulfite Sanger sequencing was conducted. As shown in Fig. 2c, *NF2* promoter methylation in GBM brain samples was increased compared with normal brain samples, and the exact methylation sites are indicated in Fig. 2d. These data suggested that methylation of NF2 might contribute to the development of GBM. This step may be importantly attributed to DNMT1, given the important role of DNMT1 in methylation.

### MiR-152-3p directly targeted DNMT1

**Fig. 3:**
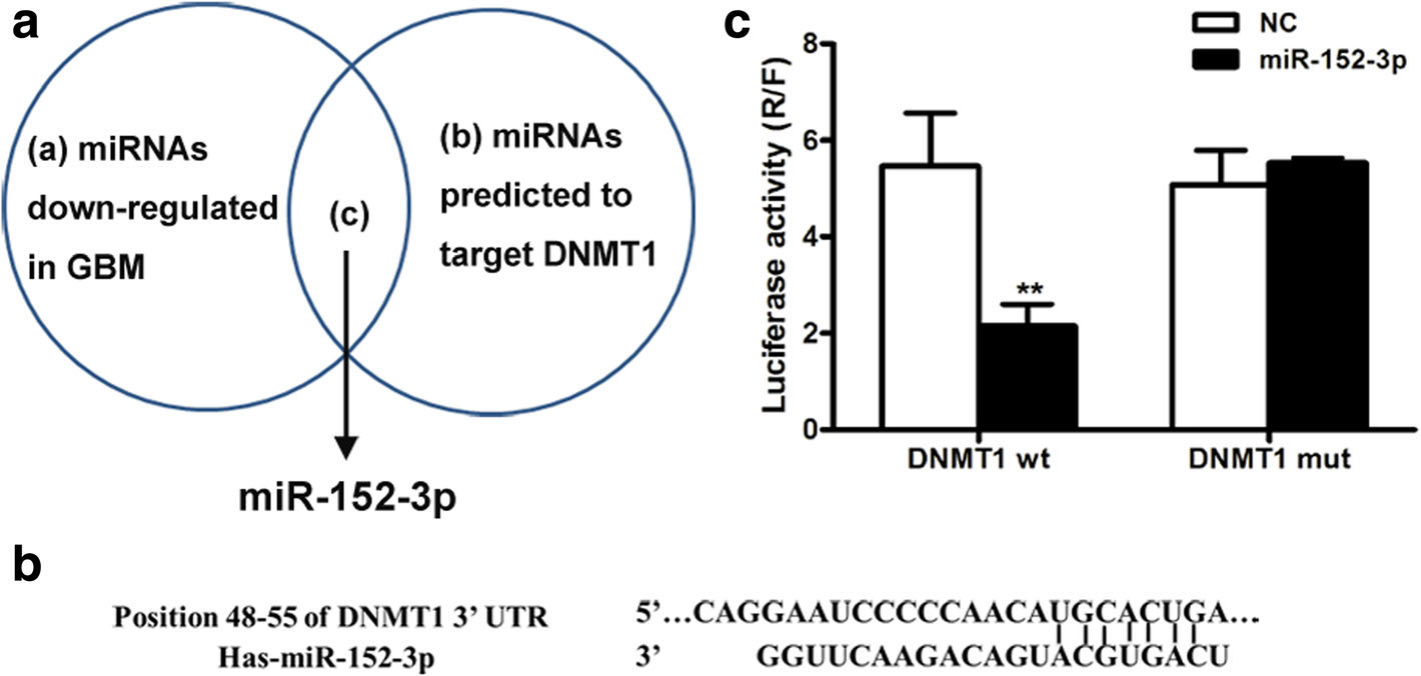
MiR-152-3p directly targets DNMT1. **a** MiR-152-3p was predicted to target REST. **b** Predicted DNMT1 3′ -UTR binding sites for miR-12-3p. Alignment of the seed regions of miR-152-3p with DNMT1 3′ UTR are shown. **c** MiR-152-3p directly targets DNMT1. A luciferase reporter vector containing either wild type (wt) or mutated (mut) DNMT1 3′-UTR was co-transfected with miR-152-3p precursor or scramble control in 293 cells, and the luciferase assay was performed. Mean and SEM of 3 independent MSC cultures are shown. **, *P* < 0.01 compared with control

MiRNAs regulate the expression of DNMT1 during the pathogenesis of GBM. It has been reported that mir-152-3p is significantly decreased in GBM. Prediction with TargetScan (http://www.targetscan.org/) showed that this miRNA might directly target DNMT1 through a potential binding site in the 3′-UTR of DNMT1 at the position 48–55 (Fig. 3a and b). Two psiCHECK2 luciferase plasmids containing DNMT1–3’UTR and DNMT1–3’UTR-MUT segments were transfected into HEK293 cells, respectively, together with miR-152-3p to confirm the correlation between mir-152-3p and DNMT1. Luciferase activity was significantly decreased in the wild type (wt)-transfected cells compared with normal controls, and was comparable to the MUT-transfected cells (Fig. 3c). These results indicated that DNMT1 was a direct target of miR-152-3p, the expression of which might affect the biological function of the target.

### DNMT1 inhibited the expression of NF2

**Fig. 4:**
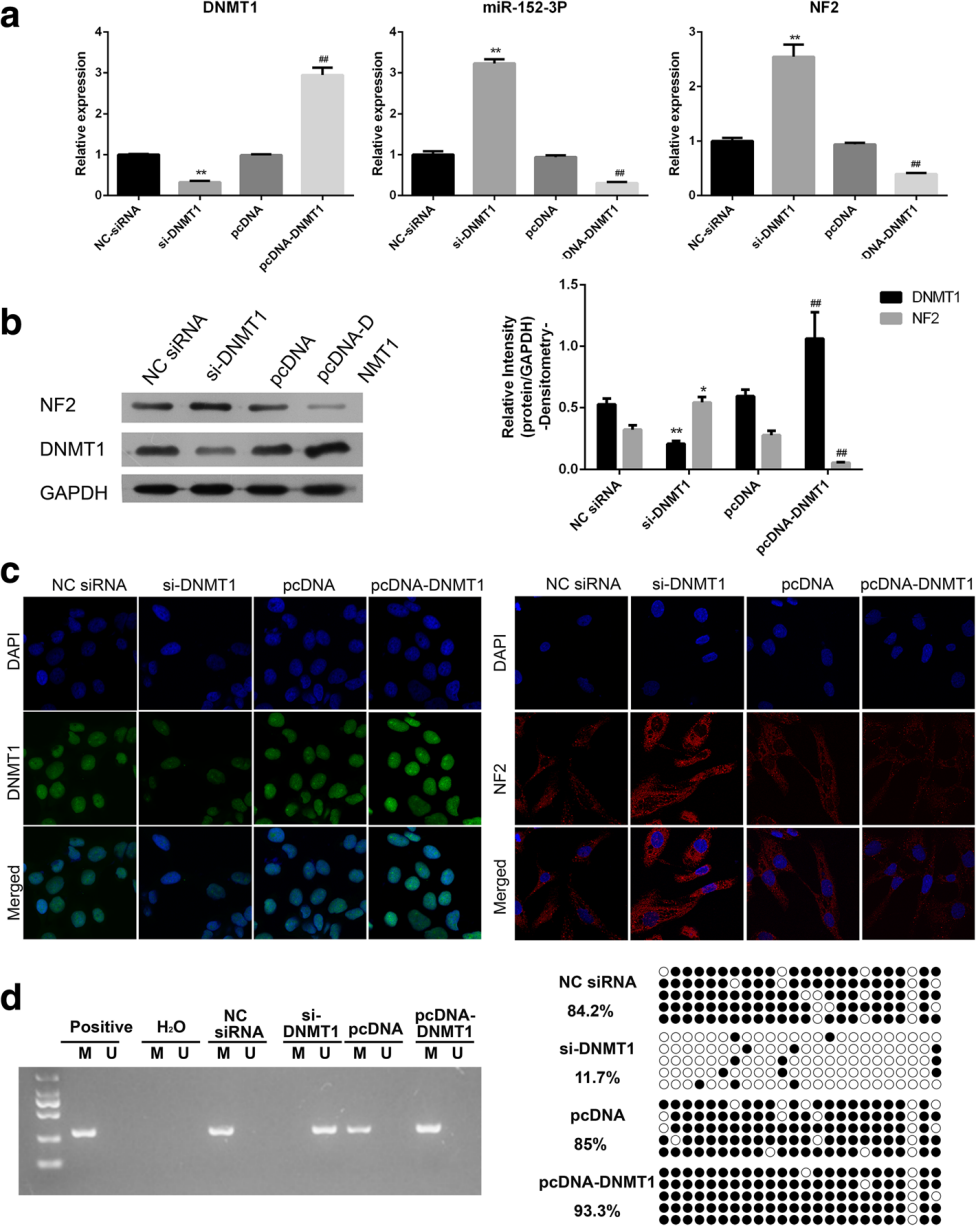
DNMT1 negatively regulates the expression of NF2. Levels of mRNA expression of DNMT1, NF2, and miR-152-3p after transfection with DNMT1 siRNA and pcDNA-DNMT1 detected by RT-PCR in U251 cells (**a**). Protein expression of DNMT1 and NF2wasdetected by Western blot (**b**) and immunofluorescence (**c**). After DNMT1 knockdown, the methylation status of NF2 in U251 cells was determined with MSP (**d**) and bisulfite Sanger sequencing (**e**). *, *P* < 0.05, **, *P* < 0.01 compared with the control groups treated with negative control (NC) siRNA. ##, *P* < 0.01 compared with control groups treated with pcDNA

In order to confirm whether DNMT1 was involved in the regulation of NF2 expression, loss of function of DNMT1 by siRNA was performed. As shown in Fig. 4a, DNMT1 mRNA expression was significantly decreased after siRNA treatment. As a result, mRNA expression of NF2 and miR-152-3p were significantly increased. Western blot and immunofluorescence showed that, in U251 cells the protein expression of DNMT1 and NF2 were significantly decreased and increased, respectively, after DNMT1 knockdown compared with controls (Fig. 4b and c). Interestingly, DNMT1 was mainly localized to the nucleus, whereas NF2 to the cytoplasm and nucleus. The role of DNMT1 in the hypermethylation of NF2 was investigated in U251 cells. As show in Fig. 4d and e, the NF2 gene was demethylated after DNMT1 knockdown. To further confirm the effect of DNMT1 on the NF2 expression, the U251 cells were subjected to the DNMT inhibitor, 5-azacytidine (5-Aza), and the expression of NF2 was detected by RT-PCR and Western blot. Consistent with the results of DNMT siRNA transfection, the expression of NF2 was significantly increased after DNMT inhibitor treatment compared with the PBS-treated group (Additional file 1 **:** Figure S1).

Moreover, in order to further confirm the role of DNMT1 in the regulation of NF2 expression, rescue experiments were performed in which pcDNA-DNMT1 was transfected in the cells. As shown in Fig. 4a and b, DNMT1 mRNA and protein expression levels were significantly increased after pcDNA-DNMT1 transfection, while the expression of NF2 andmiR-152-3p was significantly downregulated. The same pattern was also observed in the immunofluorescence analysis in U251 cells (Fig. 4c). Also, as show in Fig. 4d and e, the NF2 gene was methylated after DNMT1 overexpression. These data indicated that DNMT1 critically contributes, through methylation, to the inhibition of expression of NF2.

### MiR-152-3p increased the expression of NF2 through decreased DNMT1 expression

**Fig. 5:**
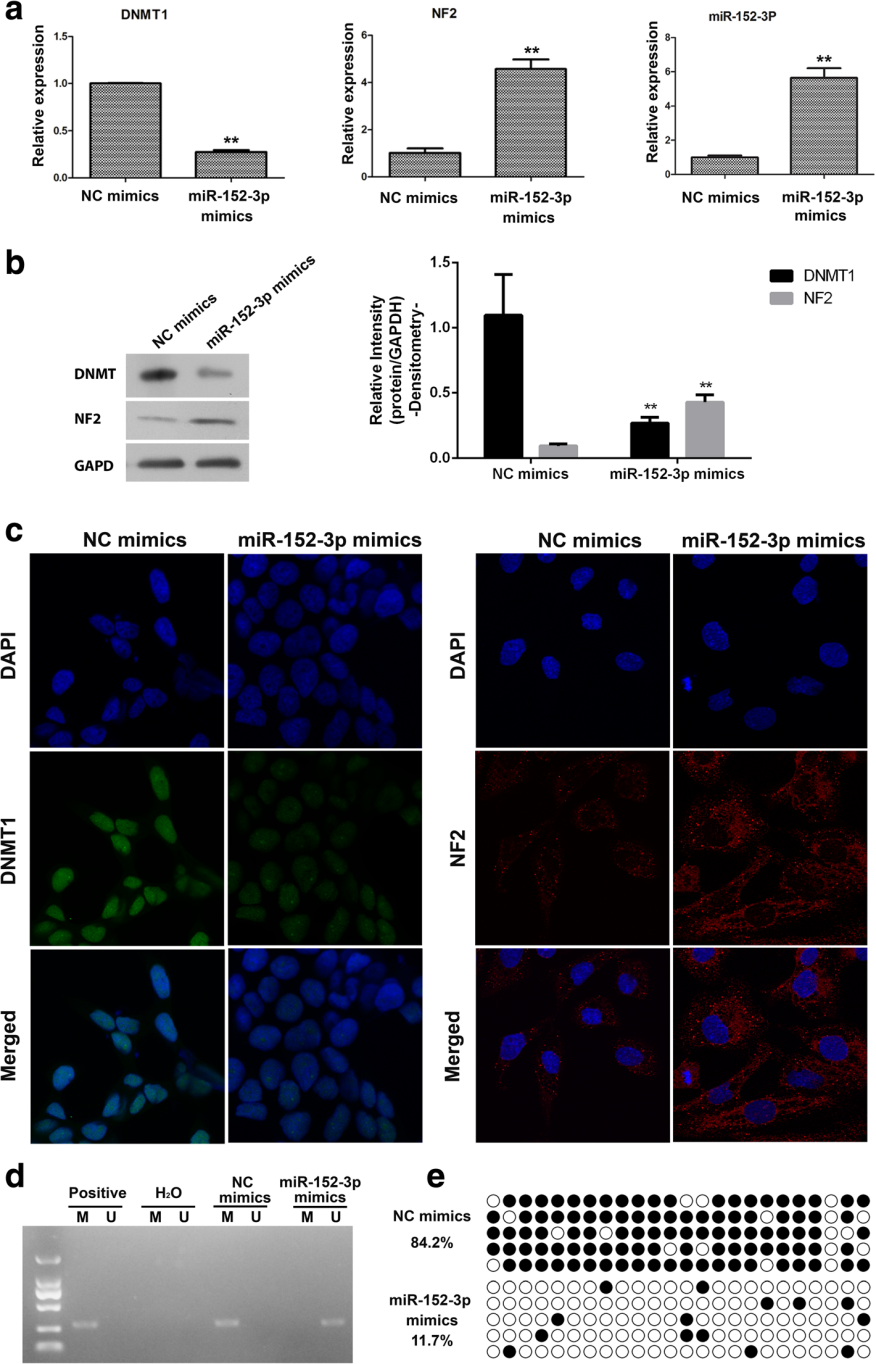
MiR-152-3p was involved in the regulation of NF2expression.mRNA expression of DNMT1, NF2, and miR-152-3p after transfection with miR-152-3p mimics, detected by RT-PCR in U251 cells (**a**). Protein expression of DNMT1 and NF2 was detected by Western blot after transfection with miR-152-3p mimics (**b**). The expression of DNMT1 and NF2in U251 cells was detected by immunofluorescence (**c**). After miR-152-3p transfection, the methylation status of NF2 in U251 cells was determined with MSP (**d**) and bisulfite Sanger sequencing (**e**). **, *P* < 0.01 compared with control groups treated with NC mimics

In order to confirm whether miR-152-3p was involved the expression of NF2, gain of function of miR-152-3p in U251 cells was performed. As shown in Fig. 5a, forced expression of miR-152-3p significantly decreasedDNMT1 mRNA expression. As a result, mRNA expression of NF2 and miR-152-3p was significantly increased. Likewise, protein expression of DNMT1 and NF2 were significantly decreased and increased, respectively, compared with controls (Fig. 5b and c). Furthermore, it was noted that miR-152-3p resulted in the demethylation of NF2 gene (Fig. 5d and e). These data indicated that miR-152-3p inhibited the expression of DNMT1, which led to upregulated expression of NF2 via demethylation.

### NF2 overexpression induced cell apoptosis and inhibited cell invasion

**Fig. 6:**
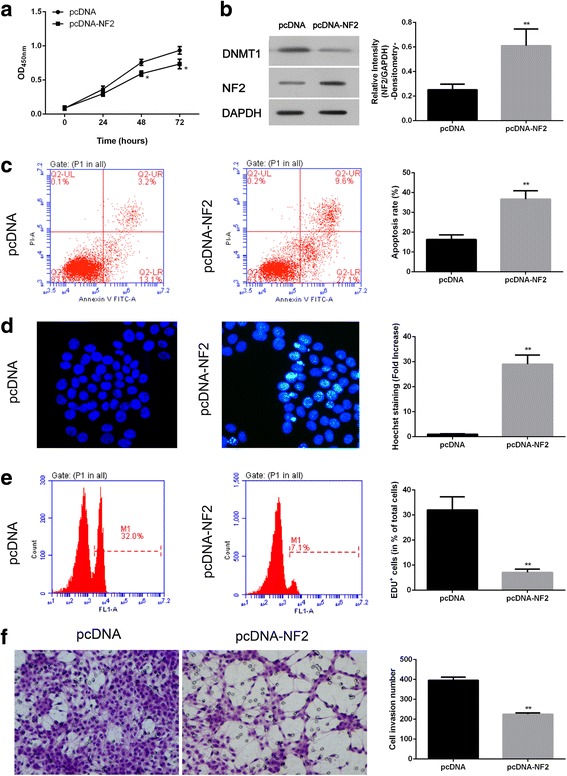
NF2 was involved in the apoptosis and invasion of U251 cells. The viability of U251 cells was determined at 24, 48 and 72 h after NF2 overexpression (**a**). Protein expression of DNMT1 and NF2 after transfection with pcDNA-NF2 was detected by Western blot in U251 cells (**b**). At 48 h after NF2 overexpression, cell apoptosis was evaluated by flow cytometry (**c**) as well as Hoechst staining (**d**), and the cell proliferation was detected with Edu (**e**); cell invasive activity was detected by transwell (**f**). *, *P* < 0.05, **, *P* < 0.01 compared with control groups

As an important tumour suppressor, NF2 plays a major role in inhibiting the proliferation of cells. As shown in Fig. 6b, Western blot showed that the expression of NF2 was significantly increased when the cell was transfected with pcDNA-NF2. The role of NF2 in the proliferation and apoptosis of cells was then investigated. We observed that NF2 overexpression significantly decreased cell viability at 48 and 72 h after transfection (Fig. 6a). Flow cytometry (Fig. 6c), Hoechst staining (Fig. 6d), and EDU assay (Fig. 6e) showed that NF2 overexpression significantly induced cell apoptosis and decreased cell proliferation at 48 h after transfection. The invasive ability of cells critically contributes to tumour development. The effect of NF2 on cell invasion was assessed. As shown in Fig. 6f, the invasion of U251 cells was significantly inhibited by NF2 overexpression compared with control groups, indicating that decreased NF2 might critically contribute to the invasion of cells in GBM.

### DNMT1 knockdown and miR-152-3p overexpression induced cell apoptosis and inhibited cell invasion

**Fig. 7:**
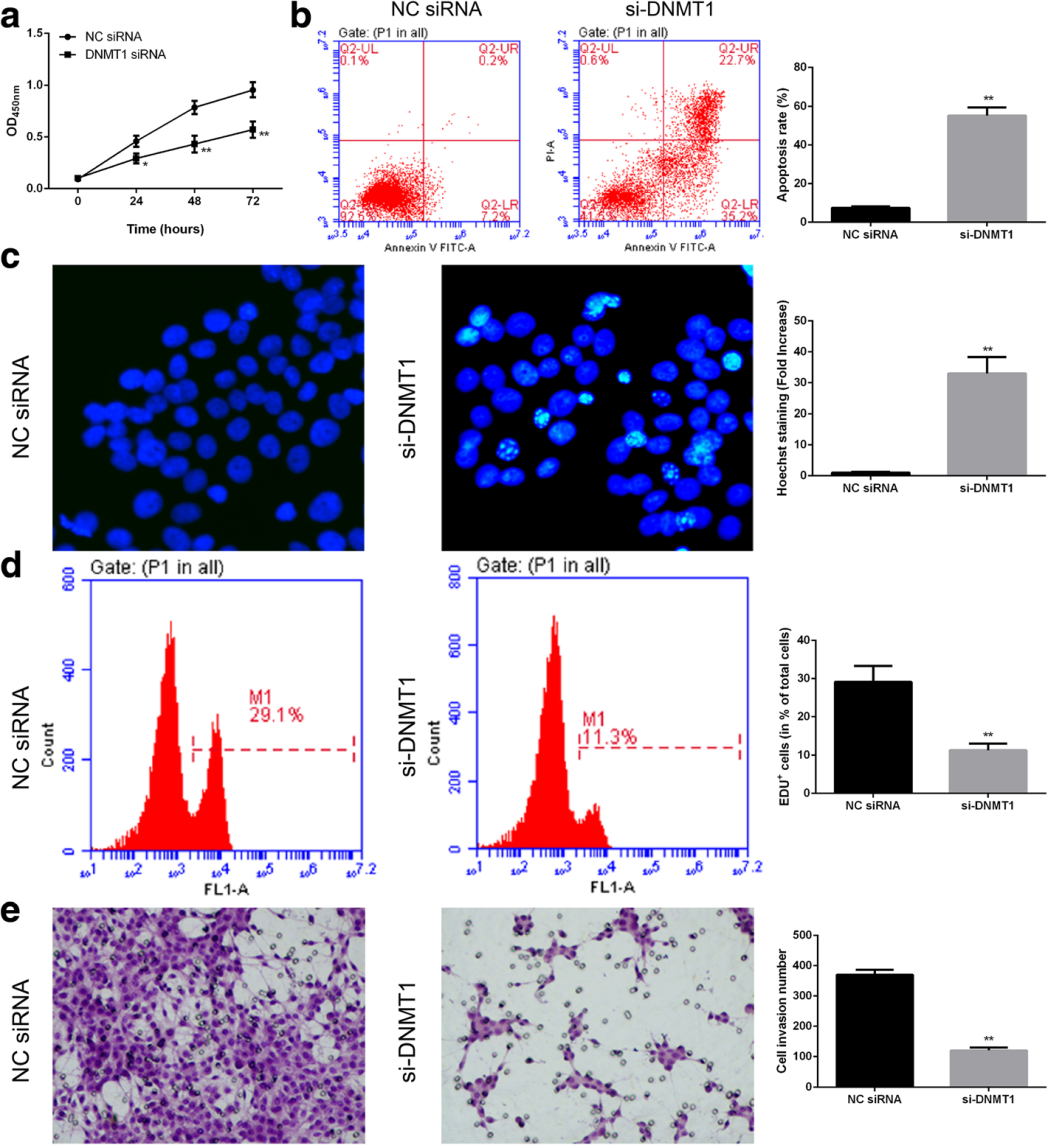
DNMT1 was involved in apoptosis and invasion of U251 cells. The viability of U251 cells was determined at 24, 48 and 72 h after DNMT1 knockdown (**a**). At 48 h after DNMT1 knockdown, cell apoptosis was evaluated by flow cytometry (**b**) as well as Hoechst staining (**c**), and cell proliferation was detected with Edu (**d**); cell invasive activity was detected by transwell (**e**). *, *P* < 0.05, **, *P* < 0.01 compared with control groups

**Fig. 8:**
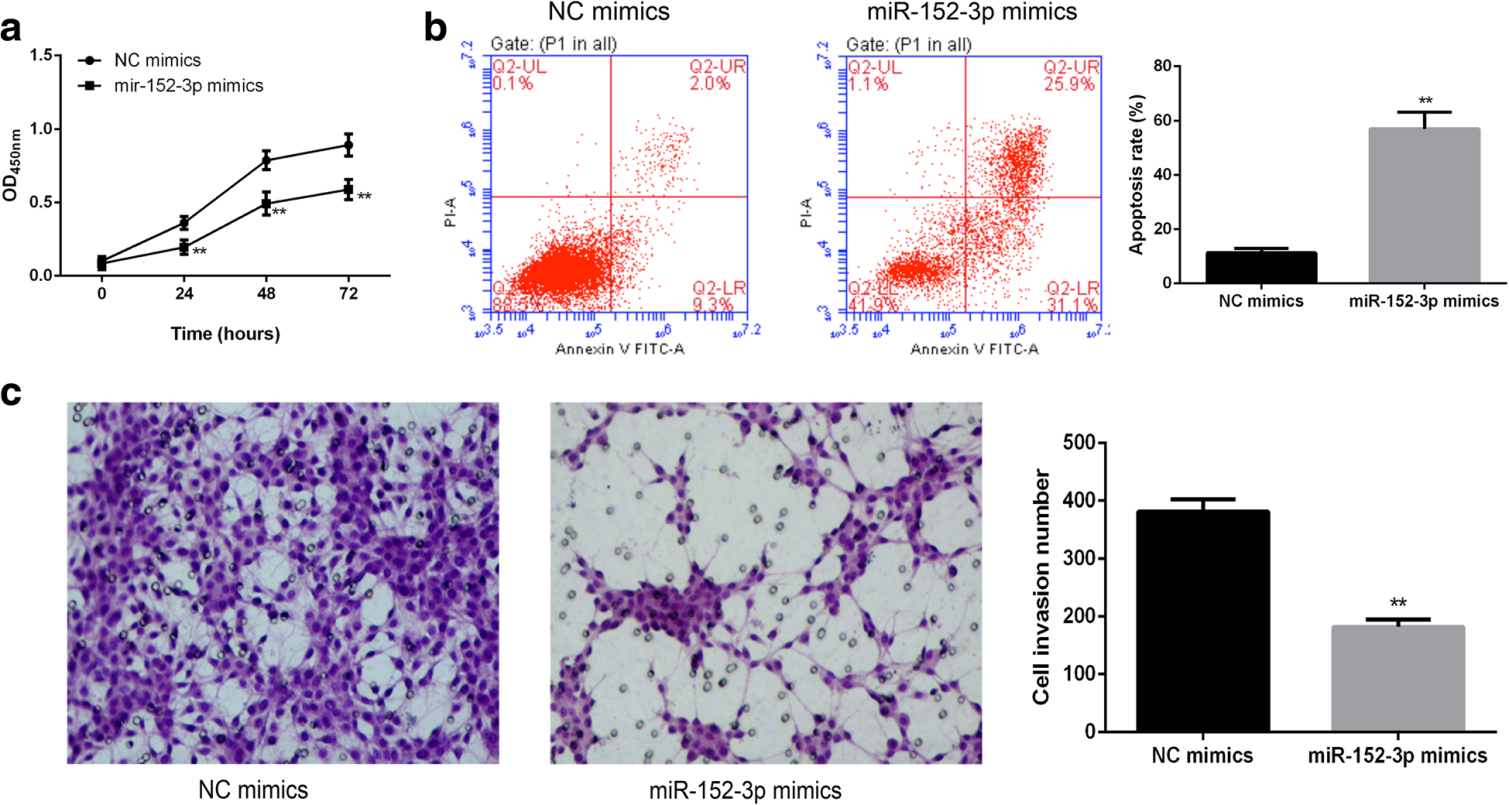
MiR-152-3p was involved in apoptosis and invasion of U251 cells. **a** The viability of U251 cells was determined at 24, 48 and 72 h after miR-152-3p overexpression. At 48 h after miR-152-3p overexpression, cell apoptosis was evaluated by flow cytometry (**b**) and cell invasive activity was detected by transwell (**c**). **, *P* < 0.01 compared with control groups

As an important direct regulator of NF2, the role of DNMT1 in cell viability was assessed. As shown in Fig. 7a, DNMT1 knockdown significantly decreased cell viability at 48 h after siRNA transfection compared with the control. Similar to the effect of NF2 overexpression, this was confirmed with the apoptosis and proliferation assays (Fig. 7b–d). Additionally, DNMT1 knockdown inhibited cell invasion (Fig. 7e). These results indicated that DNMT1 might critically contribute to the cell proliferation and invasion by inhibiting NF2 expression. Subsequently, the effect of miR-152-3p on cell viability was determined. As expected, miR-152-3p overexpression significantly decreased cell viability compared with control treatment (Fig. 8a). Additionally, we observed that miR-152-3p overexpression significantly increased cell apoptosis and decreased the cell invasion compared with the control (Fig. 8b and c).

## Discussion

GBM is the most frequent and aggressive type of glioma. Its high rate of recurrence and resistance to chemotherapeutics predicts a poor prognosis, and highlights the critical need to identify an effective target for therapy. GBM can be induced by different mechanisms, as shown by transcriptional and proteomic. Inactivation and downregulation of the tumour suppressor NF2 has been widely recognized as a critical contributor to GBM. The goal of this study was to explore the underlying mechanism of regulation of the expression of this protein. In this study we showed for the first time that miRNA 152-3p mediates NF2 gene methylation via DNMT1, which critically contributes to the development of GBM. Our results further demonstrate that DNMT1 and miR-152-3p form a negative feedback loop, while NF2 and miR-152-3p form a positive feedback loop.

NF2 is a FERM family protein and plays a critical role in the establishment of adherent junctions and is an important mediator of contact inhibition [15]. It is involved in the signalling pathways regulating cell-matrix adhesion, cell proliferation, survival and invasiveness [16]. It has been reported that NF2 activity is regulated by multiple mechanisms. In this study, NF2 was confirmed to be significantly downregulated in the GBM, while DNMT1 was markedly upregulated. Furthermore, we observed hypermethylation of NF2 in GBM tissues, a finding that has not been previously reported. U251, U87, T98-G and A172 are human glioma cells that are routinely used in vitro studies of GBM. Consistent with findings in GBM tumour tissues, NF2 and DNMT1 levels were decreased and increased, respectively, in these cells. Because the contrast was greatest in U251 cells, that cell line was chosen for investigating the correlation between DNMT1 and GBM. Furthermore, because hypermethylation of NF2 was noted in U251 cells, that cell line was also used for further investigation.

The present study showed that in U251 cells, DNMT1 plays a critical role in the proliferation and invasion of GBM cells by decreasing NF2 expression. DNMT1, the maintenance methyltransferase, is widely expressed, particularly in the brain, where there is high expression, and is frequently upregulated in various human cancers such as colon, endometrioid, and prostate [17–19]. Additionally, DNMT1 decreases hTERT expression by hypermethylation, resulting in its reduced expression in glioma cells andenhancedgliomachemosensitivity [20]. ADAMTS9-AS2, a novel tumour suppressor, was modulated by DNMT1 and subsequently contributed to glioma development [21]. Together with the well-recognized role of NF2 in glial cell proliferation in some human malignant gliomas, these findings indicated that increased expression of DNMT1 might promote the development of GBM by decreasing the expression of NF2 through methylation.

It is widely recognized that aberrant miRNAs are involved in various pathogenetic processes [22, 23]. Furthermore, accumulating evidence suggests the presence of different miRNAs with anti-oncogenic properties in glioblastomas [24]. An important mechanism underlying this is their contribution to aberrant DNA hypermethylation through regulation of DNMTs. miR-152 is one of the miRNAs that has attracted considerable interest in recent years, since it is implicated in glioma and other types of cancer [25, 26]. It targets the 3′-UTR of DNMT1, resulting a significant decrease in DNMT1 at both the mRNA and protein levels. This was confirmed in studies on ovarian cancer, breast cancer, pancreatic cancer and prostate cancer [13, 27–29]. MiR-152-3p, one of the two mature miR-152 sequences, shares the same seed sequence of approximately 6–7 nucleotides with the other members of miR-152 family; the aberrant expression of miR-152-3p was reported to be related to the pathogenesis of tumours such as hepatitis B virus-related hepatocellular carcinoma [13, 30]. In the present study, we showed that miR-152-3p directly targeted DNMT1. We also postulate that overexpression of miR-152-3p significantly enhances demethylation, which further upregulates the expression of NF2. Functionally, miR-152-3p overexpression, DNMT1 knockdown and NF2 overexpression significantly induced glioma cell apoptosis and inhibited their invasion. These data indicated that miR-152-3p might play an important role in GBM suppression via DNMT1-mediated downregulation of NF2.

## Conclusion

In conclusion, our results suggest that miRNA-152-3p functions as a novel regulator to promote glioma cells invasion via DNMT1-mediated downregulation of NF2 and can potentially be used as a treatment for GBM.

## Additional file

Additional file 1: Figure S1. The DNMT inhibitors 5-Azareverses the inhibitory effect of DNMT1 on NF2 expression. Protein and mRNA expression of NF2 in U251 cells after treatment with 5-Aza was detected by RT-PCR (A) and Western blot (B). **, *P* < 0.01 compared with the PBS-treated control group. (TIFF 12918 kb)

## Abbreviations

CCK-8: Cell-Counting Kit-8
DNMTs: DNA methyltransferases
FDA: Food and Drug Administration
GBM: Glioblastoma multiforme
IHC: Immunohistochemistry
miRNAs: MicroRNAs
mRNA: messenger RNA
MSP: Methylation specific PCR
NC: Negative control
NF2: Neurofibromatosis type 2
PI: Propidium iodide
qRT-PCR: Quantitative real-time PCR
