# Supplementary figures and images for: Regulation of human glioma cell apoptosis and invasion by miR-152-3p through targeting DNMT1 and regulating NF2: MiR-152-3p regulate glioma cell apoptosis and invasion
Source: J Exp Clin Cancer Res. 2017 Aug 1;36:100. doi: 10.1186/s13046-017-0567-4 (PMC5539621; doi:10.1186/s13046-017-0567-4)

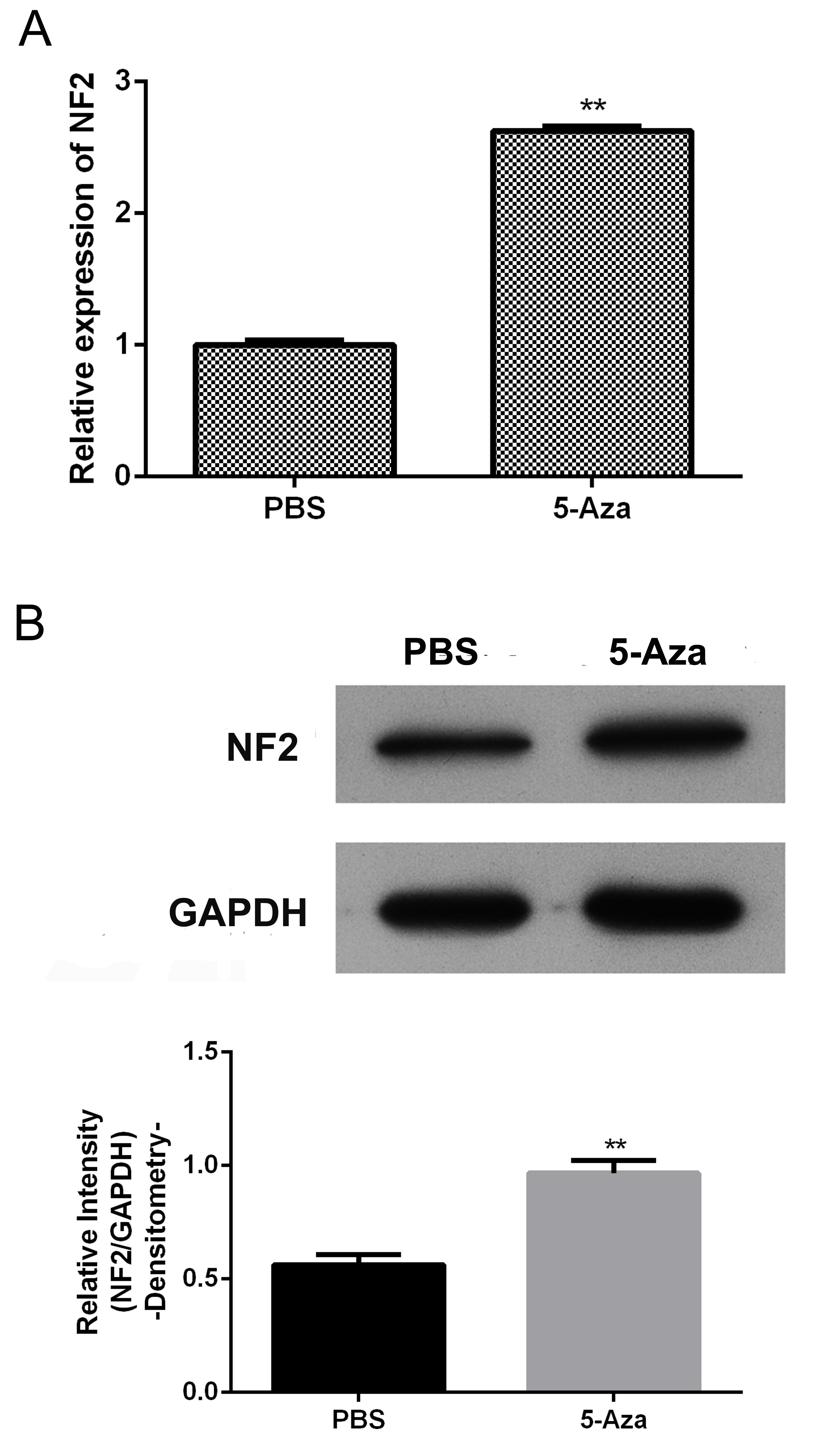

Supplement: Additional file 1: Figure S1. — The DNMT inhibitors 5-Azareverses the inhibitory effect of DNMT1 on NF2 expression. Protein and mRNA expression of NF2 in U251 cells after treatment with 5-Aza was detected by RT-PCR (A) and Western blot (B). **, P < 0.01 compared with the PBS-treated control group. (TIFF 12918 kb) [file 13046_2017_567_MOESM1_ESM.tif]
